# From Scalpel to Scope: How Surgical Techniques Made Way for State-of-The-Art Endoscopic Procedures

**DOI:** 10.1016/j.gastha.2023.10.013

**Published:** 2023-11-04

**Authors:** Firas Bahdi, Amanda Labora, Sagar Shah, Maryam Farooq, Peerapol Wangrattanapranee, Timothy Donahue, Danny Issa

**Affiliations:** 1 Division of Digestive Diseases, Department of Medicine, David Geffen School of Medicine, University of California, Los Angeles, Los Angeles, California; 2 Department of Surgery, David Geffen School of Medicine, University of California, Los Angeles, Los Angeles, California; 3 David Geffen School of Medicine, University of California, Los Angeles, Los Angeles, California; 4 Department of Medicine, Keck School of Medicine of the University of Southern California, Los Angeles, California

**Keywords:** Therapeutic Endoscopy, Endoscopic Innovation, Minimally-Invasive, Surgical Endoscopy

## Abstract

The continuous evolution of endoscopic tools over the years has paved the way for minimally invasive alternatives to surgical procedures for multiple gastrointestinal conditions. While few endoscopic techniques have supplanted their surgical counterparts like percutaneous gastrostomy tubes, many have emerged as noninferior, less morbid alternatives for such diverse conditions as achalasia (peroral endoscopic myotomy), obesity (endoscopic sleeve gastroplasty), drainage of pancreatic walled off necrosis (EUS-guided cystogastrostomy), and gastric outlet obstruction (EUS-guided gastrojejunostomy). These techniques were based on surgical concepts and would not have been feasible without collaboration between surgeons and endoscopists. Such collaboration is exemplified by the antireflux fundoplication, which features combined hiatal hernia repair with transoral and incisionless fundoplication. The burgeoning armamentarium of endoscopic alternatives to traditional surgical procedures requires a multidisciplinary discussion and individually tailored treatment plans that consider patient preferences as well as the relative risks and benefits of surgical and endoscopic approaches. As technological advances give rise to ever more innovative endoscopic techniques, studies to evaluate clinical outcomes and define their role in treatment algorithms will be required.

## Introduction

Advances in endoscopic procedures over the past several decades have ushered in minimally invasive therapies for a wide range of gastrointestinal conditions. Even the most complex conditions have a contemporary endoscopic approach that provides at least a noninferior alternative to surgery. These developments resulted from the endless efforts of innovators aided by technological advances and inspiration by surgical techniques. Building on surgical principles and armed with smarter and less expensive tools, endoscopists and surgeons collaborated to develop innovative, minimally invasive approaches to surgical procedures. In this review, we describe the main endoscopic interventions utilized today and highlight their surgical roots (Table). These interventions include gastrostomy tube placement, lower esophageal sphincter myotomy, Zenker’s diverticulum (ZD) treatment, antireflux fundoplication, restrictive gastric bariatric intervention, debridement of pancreatic walled-off necrosis, and bypass gastrojejunostomy.

**Table:** Summary Table of Discussed Conditions and Their Relevant Surgical & Endoscopic Approaches

Condition	Pathogenesis	Original concept	Surgical approach	Endoscopic approach	Modified concept
Dysphagia	Oropharyngeal or esophageal dysphagia	Placing a feeding tube into the stomach to bypass the oropharynx & esophagus	Surgical gastrostomy tube placement	Percutaneous endoscopic gastrostomy tube placement	Endoscopic insufflation to approximate the stomach to abdominal wall for intragastric trocar introduction and subsequent peroral pull of gastrostomy tube while avoiding laparoscopy
GERD	Malfunction of the antireflux barrier (LES sphincter, crural diaphragm, and angle of his)	Restoration of antireflux barrier but plicating the gastric fundus around the distal esophagus and reducing hiatal hernia	Surgical fundoplication	Transoral incisionless fundoplication	Create an approximately 270 degrees warp around the distal esophagus by pulling the gastric fundus down and around it using polypropylene fasteners
Obesity	Multifactorial	Reducing gastric volume and inducing delayed emptying by removing the greater curve & fundus surgically and stapling the rest of the stomach in a sleeve-like form.	Laparoscopic sleeve gastrectomy	Endoscopic sleeve gastroplasty	Performing full thickness plication to reduce gastric volume in an endoscopic, incisionless, approach while avoiding resecting parts of the stomach
Achalasia	Degeneration of neurons in LES resulting in failure of relaxation and loss of peristalsis	Relieving resting pressure at the LES by cutting the muscle fibers	Laparoscopic myotomy	POEM	Creating a tunnel through submucosa and performing myotomy endoscopically with the benefit of extended myotomy for type 2 and 3 achalasia
Zenker’s diverticulum	Outpouching of the esophagus between the CP muscle and the inferior pharyngeal constrictor muscle	Preventing food impaction in the diverticulum by resection or dissecting the muscular septum between the esophagus and diverticulum	Surgical diverticulectomy	Z-POEM	Creating a submucosa tunnel to dissect the muscular septum and extending it on both the diverticular and esophageal side using flexible endoscopy
Pancreatic walled off necrosis	Collection of necrotic pancreatic tissue as a sequela of severe pancreatitis	Drainage of necrotic collection	Videoscopic assisted retroperitoneal debridement or laparoscopic debridement or laparoscopic cystogastrostomy	EUS-guided cystogastrostomy	Placement of an EUS-guided lumen apposing metal stent across the stomach into the necrotic pancreatic collection for internal drainage and necrosectomy
Gastric outlet obstruction	Obstruction of the gastric outlet and/or duodenum	Bypassing the area of obstruction by connecting the stomach to the jejunum	Surgical gastrojejunostomy	EUS-guided gastrojejunostomy	Placement of an EUS-guided lumen apposing metal stent across the stomach into the jejunum to bypass the area of obstruction

For each of these interventions, we will review the pathogenesis of the disease it targets and describe the standard surgical approach, emphasizing the key surgical concept that made it successful. Furthermore, we will examine the transition made over the years to reach the contemporary endoscopic approach. Finally, we will present an illustration to accompany each procedure to compare the surgical and endoscopic approaches.

## Techniques

### Surgical Gastrostomy Tube → Percutaneous Endoscopic Gastrostomy (PEG) Tube

**Figure 1:**
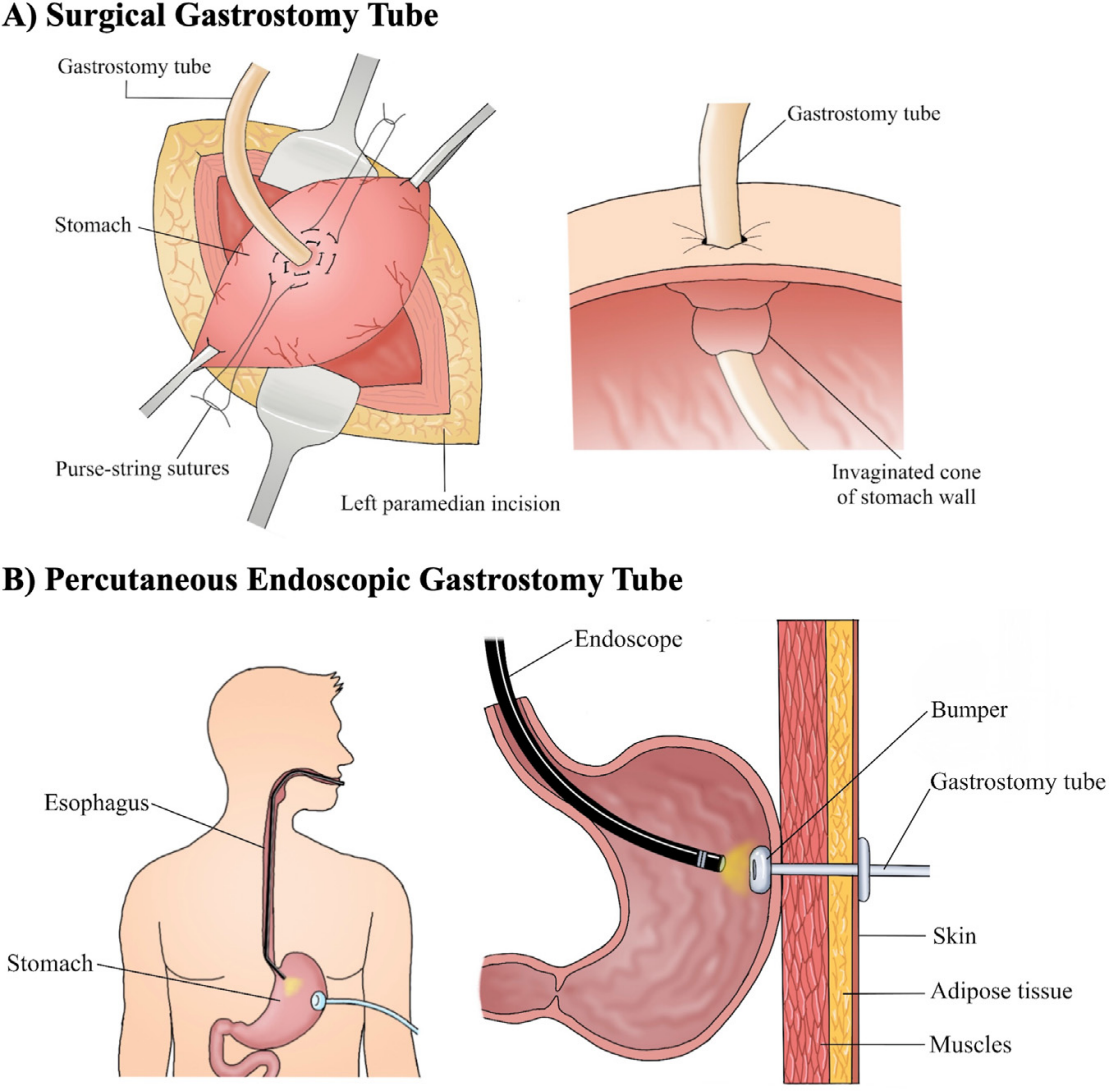
Illustration of surgical gastrostomy tube insertion (A) vs percutaneous endoscopic gastrostomy tube placement (B).

In June 1979, a 4.5-month-old infant was referred to pediatric surgeon Dr Michael Gauderer at University Hospitals of Case Western Reserve University in Cleveland for a surgical gastrostomy tube (Figure 1).^1^^,^^2^ The infant was suffering from a neurological disease that prohibited oral intake. At University Hospitals, Dr Jeffrey Ponsky, a young surgeon and a skilled endoscopist, was consulted. He had been thinking about a less invasive way to achieve enteral feeding for some time. It was then when the first percutaneous endoscopic gastrostomy (PEG) tube placement was performed by Dr Ponsky.^1^ The initial attempts following that date allowed for technique improvements and decreased complications. Today, over 250,000 PEGs are done every year in the United States.

#### Original surgical concept

##### 1- Open surgical gastrostomy tube

Following induction of general anesthesia, an open gastrostomy tube placement begins with an incision over the left rectus sheath approximately 2 cm below the left costal margin (Figure 1A). This is carried down through the subcutaneous tissues and abdominal wall muscles using electrocautery.^3^, ^4^, ^5^ The abdominal cavity is accessed by incising the peritoneum. The liver is retracted superiorly to reveal the stomach below, which is then brought up through surgical incision. Two circular purse string sutures are placed in the anterior wall of the stomach using 3-0 Vicryl.^3^ A gastrotomy is made using electrocautery and a 12-French catheter is placed into the stomach. The purse strings are then tied around the catheter and the stomach is approximated to the abdominal wall. The peritoneum is closed followed by approximation of the fascial, deep dermal, and subcuticular layers. Finally, the gastrostomy tube is secured to the abdominal wall.

#### Current endoscopic approach: (pull-type PEG tube)

The patient is placed in supine position. The stomach is insufflated to oppose gastric and abdominal walls (Figure 1B).^2^^,^^6^ A site is identified in the body of the stomach with good transillumination for placement, usually on the anterior wall of the distal body. The abdominal wall is marked and prepped in a sterile manner. The trocar needle is introduced through the abdominal wall and into the stomach under direct endoscopic view. A snare is introduced through the endoscope to grab a guide wire that is passed through the trocar. The endoscope and snare are removed, pulling the wire out through the mouth. A skin incision is made at the site of needle insertion. The gastrostomy tube is lubricated, attached to the wire, and pulled through the mouth and into the stomach. The trocar is removed, and the gastrostomy tube is pulled out from the stomach through the skin. The endoscope is reinserted to confirm the appropriate position of the balloon/internal bumper. The external bumper is passed over the external part of the tube, along with the clamp and the feeding attachment. The final tension and compression of the abdominal wall by the PEG tube and external bumper are checked and should allow the bumper to touch the skin lightly, and the PEG balloon/internal bumper lightly touching the stomach wall. Topical antibiotics are applied around the gastrostomy skin site. The feeding tube is capped, and the site is cleaned and dressed.

#### Comparisons

The percutaneous endoscopic approach is faster (average procedure duration 30 minutes) compared to the laparoscopic approach (procedure time 48 minutes) and open approach (procedure time 68 minutes).^7^ PEG tube placement is associated with lower cost and morbidity. The technique is relatively safe and allows for a quick resumption of enteral feeding, as early as 4 hours after PEG tube insertion. In a recent study by Kohli et al. a total of 180,068 patients were analyzed using a national readmission database.^8^ The risk for colon perforation was higher with interventional radiology (IR) guided gastrostomy tube (relative risk [RR] = 1.9) and the surgical approach (RR = 6.6) compared to the endoscopic approach (*P* < .001). Similarly, the odds for infection were higher with the IR approach (RR = 1.3) and the surgical approach (RR = 1.6). Furthermore, the inpatient mortality rate on multivariate analysis was 1.5% for the surgical approach and 1.09% for IR approach vs endoscopic gastrostomy tube placement (*P* = .02).

### Surgical Fundoplication → Transoral Incisionless Fundoplication (TIF)

**Figure 2:**
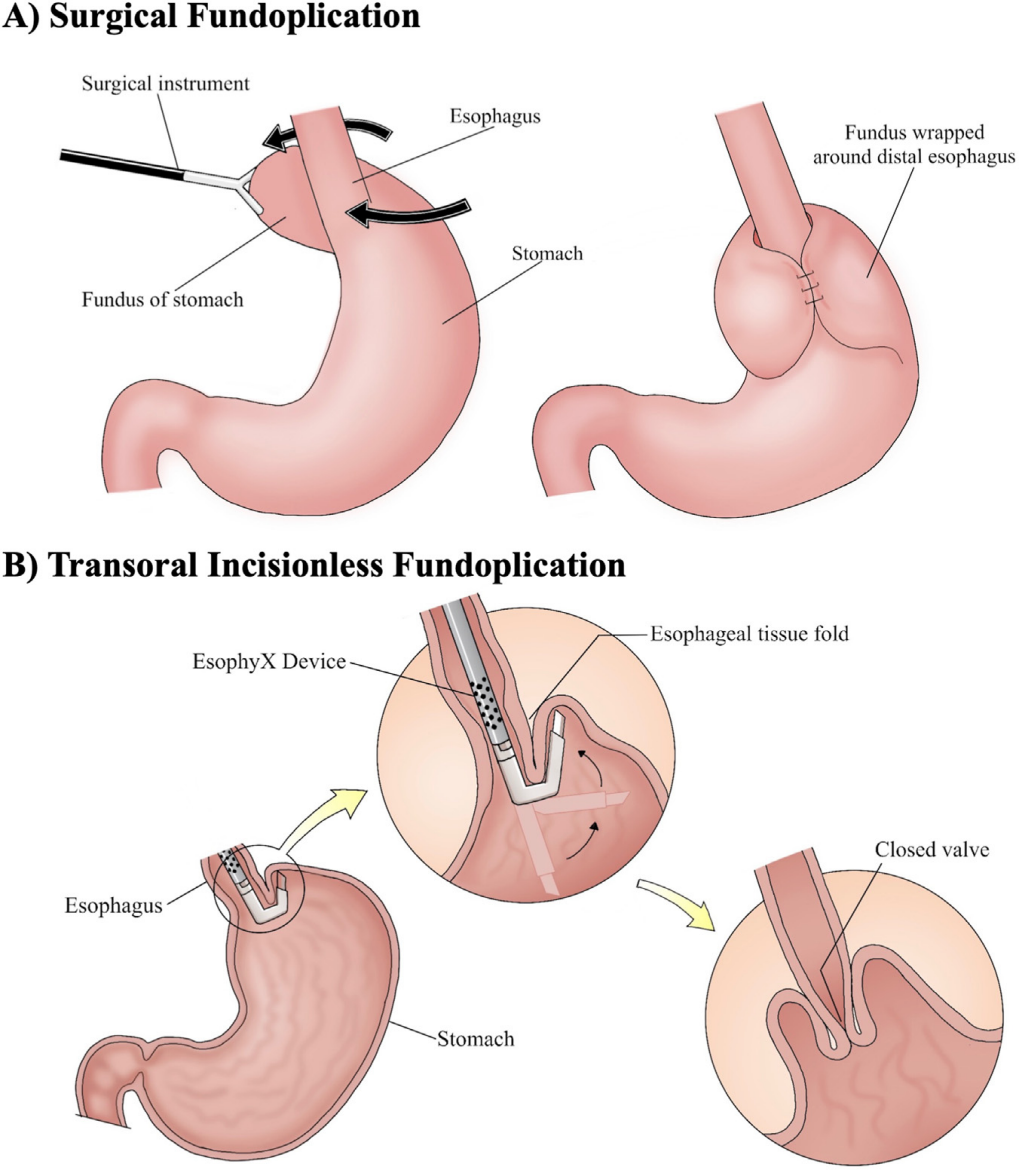
Illustration of surgical fundoplication (A) vs transoral incisionless fundoplication (B).

Gastroesophageal reflux disease (GERD) is caused by the retrograde movement of acidic gastric contents into the esophagus (Figure 2). The antireflux barrier at the esophagogastric junction (EGJ) protects the esophagus from these corrosive contents and is comprised of the lower esophageal sphincter (LES) and the crural diaphragm. The LES is a 3–4 cm segment of tonically contracted muscular fibers that maintain intraesophageal pressures greater than in intragastric pressures, thereby preventing reflux. Physiologic triggers, like swallowing, can result in depressurization of these structures, allowing food boluses to pass into the stomach. Other triggers can increase the frequency of transient LES relaxations and pathogenic reflux. These triggers include smoking, caffeine, pregnancy, and certain medications.^9^ Additionally, the gastric sling fibers that run along the anterior and posterior walls of the fundus play a crucial role in the maintenance of the angle of His; disruption of this angle is also implicated in the pathogenesis of GERD.

The earliest surgical interventions were focused on correction of anatomical defects. The Allison repair attempted to reestablish the normal anatomy of the crural sling fibers in addition to reducing any hiatal hernia and returning the esophageal hiatus to its normal width.^10^^,^^11^ However, recurrence of hernias and symptoms using this technique was common. In fact, Nissen observed symptomatic and anatomic recurrence rates near 50% following Allison repair.^12^ Nissen drew on his experience with a patient whose reflux symptoms had resolved after Nissen resected an esophageal ulcer and then affixed the distal esophagus to the anterior wall of the gastric body to seal the anastomosis and prevent leakage.^13^ Nissen fine-tuned this technique to give rise to the antireflux surgeries performed today. As innovations in surgical technology emerged, the laparoscopic Nissen fundoplication (LNF) is now the most common minimally invasive antireflux surgery. Endoscopic treatment modalities developed as an extension of this paradigm shift toward safer, less morbid procedures. The transoral incisionless fundoplication forms the basis of endoscopic treatment modalities used today.

#### Laparoscopic Nissen fundoplication (LNF)

Following induction of general anesthesia, the patient is positioned and pneumoperitoneum is achieved with one of several techniques including Hasson (open) or Veress (closed) after which the following laparoscopic ports are placed: 5-mm or 10-mm camera just left of the umbilicus, 2 5-mm ports in the right mid abdomen and an additional 5 mm port in the left subcostal area (Figure 2A).^14^ An additional 5 mm port may be placed in the subxiphoid position if a laparoscopic retractor is needed for the left lobe of the liver. Utilizing traction of the fat pad at the gastroesophageal junction, the phrenogastric ligament is exposed and divided. The lesser sac is entered by dividing the greater omentum just outside the gastroepiploic arcade and the short gastric vessels are divided to expose the left crus. To facilitate dissection of the right crus, the gastrohepatic ligament is divided, taking care to identify and avoid injury to an aberrant left hepatic artery if present. The right phrenoesophageal membrane is then incised to expose the underlying right crural fibers. Once the crural fibers are exposed, a Penrose drain is wrapped around the esophagus at the level of the hiatus and used to retract the esophagus caudally. The esophagus is extensively mobilized to ensure that at least 3 centimeters of esophagus resides within the abdominal cavity without traction. The crura are then reapproximated posteriorly, often using a 52 to 60 French bougie to guide wrap sizing. The wrap is created by passing the posterior fundus behind the esophagus where it is joined with the anterior fundus. These structures are secured together using 3 to 4 sutures over a length of 3 cm. The wrap is then secured to the diaphragm to ensure it does not herniate into the mediastinum. Patients gradually advance from clear liquids to soft foods, then resume a regular diet 4–6 weeks following surgery.^15^

#### Transoral incisionless fundoplication (TIF)

During the TIF procedure, an endoscope fitted with the single-use EsophyX Z+ device (EndoGastric Solutions, Inc.) is used to pull the gastric fundus down and around the distal esophagus, where it is anchored with polypropylene fasteners (Figure 2B).^16^ The wrap, typically 200–300 degrees, augments the crural sling fibers and steepens the angle of His. Critically, while surgical treatment addresses both LES incompetency and herniation of gastric contents into the thoracic cavity through the esophageal hiatus, TIF is most appropriate for patient with small or absent hiatal hernias; Hill grade 1 or 2 is acceptable for TIF alone, but patient’s with Hill 3 and 4 anatomy or axial hernia lengths of > 2 cm generally require concomitant hiatal hernia repair to prevent herniation of the entire wrap through the esophageal hiatus.^17^

#### Comparison

Observational data for TIF are promising. In one multicenter registry study of 100 patients undergoing TIF, 80% remained off proton pump inhibitors (PPI) at 6-month follow-up.^18^ Additionally, 73% and 65% of patients reported normalization of quality-of-life scores at 6 months and 24 months, respectively.^18^^,^^19^ While randomized data demonstrate superiority of TIF to sham or PPI in terms of quality-of-life and esophageal pH, head-to-head data for TIF and surgical antireflux procedures is lacking.^20^, ^21^, ^22^ One, small (n = 20) nonrandomized comparative, prospective study comparing TIF with LNF showed a higher prevalence of reflux esophagitis and more acid exposure in the TIF cohort over a 3-month follow-up period.^23^ A network meta-analysis utilizing studies comparing surgical and endoscopic interventions to PPI to produce an indirect comparison of the procedural techniques reported greater improvement in physiologic parameters of GERD with LNF and better health-related quality of life with TIF^24^; however, several methodologic concerns with this meta-analysis have been reported in the literature.^25^

### Laparoscopic Sleeve Gastrectomy (LSG) → Endoscopic Sleeve Gastroplasty (ESG)

**Figure 3:**
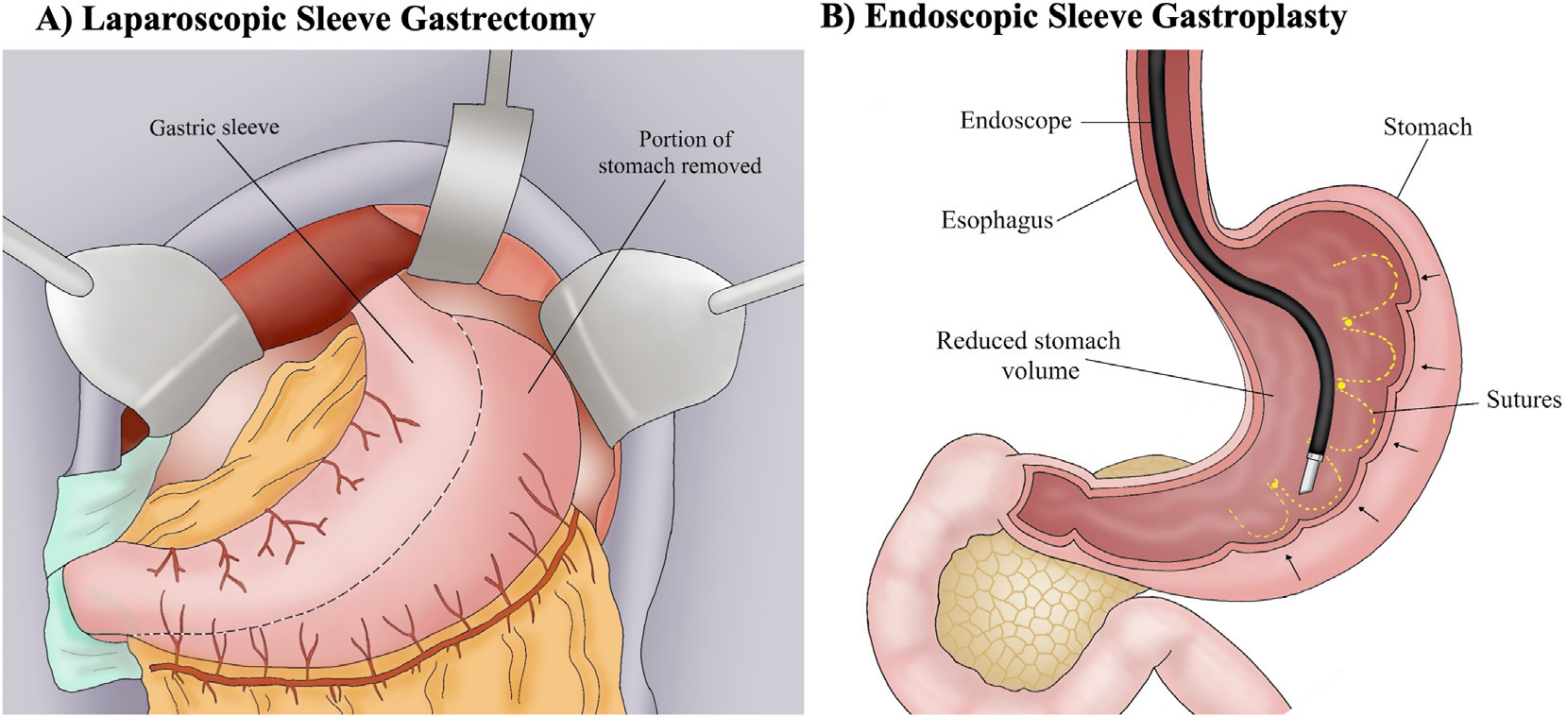
Illustration of Surgical sleeve gastrectomy (A) vs endoscopic sleeve gastroplasty (B).

The gastrointestinal tract plays a major role in the pathophysiology of obesity through stimulation of fullness & satiety, nutrient absorption, and neurohormonal feedback that orchestrates metabolism and gastric emptying (Figure 3).^26^ Various bariatric interventions have been developed over decades targeting one or more of these areas with variable success.^27^ The stomach has been a commonly identified target for bariatric interventions given its important role balancing hunger and satiety through secretion of the hormone ghrelin, mechanical fullness signals, and the rate of gastric emptying.^28^ Surgical sleeve gastrectomy involves gastric resection along the greater curvature resulting in a narrower tubular stomach. It was first described in 1988 by Dr Hess, a U.S. surgeon from Ohio, who performed open sleeve gastrectomy as a restrictive component of the biliopancreatic diversion and duodenal switch procedure, which augmented the latter’s efficacy in terms of weight loss and diabetes control.^29^ As surgical techniques improved, laparoscopic sleeve gastrectomy (LSG) became a widely performed primary bariatric surgery.^27^ The first role of endoscopy was in the management of gastric leaks after LSG by endoscopic placement of self-expanding metal stents.^30^^,^^31^ Endoscopic suturing was first utilized to address weight gain following Roux-en-Y gastric bypass in 2006 at Brigham and Women’s Hospital, where it was successfully applied to reduce a dilated gastrojejunal anastomosis.^32^ In 2011, the TRIM trial by Cleveland Clinic and Brigham & Women’s Hospital was published. This was the first pilot study highlighting the success of transoral gastric volume reduction using an endoscopic suturing system, which would go on to become known as the endoscopic sleeve gastroplasty (ESG).^33^ Since then, ESG has been optimized with excellent outcomes reported by the MERIT trial, a randomized multicenter U.S.-based trial that led to ESG approval by the Food and Drug Administration.^34^ Today, hundreds of ESG cases are performed annually across the world.

#### Laparoscopic sleeve gastrectomy (LSG)

Laparoscopic sleeve gastrectomy (LSG) indications include obesity class III (body mass index (BMI) ≥ 40 Kg/m^2^) or obesity class II (BMI 35–39.9 Kg/m^2^) with obesity-related metabolic comorbidities (Figure 3A).^27^ After induction of general anesthesia, the patient is placed in the supine position and an orogastric tube is placed.^27^^,^^35^ Pneumoperitoneum is established followed by the placement of 5 trocars.^35^ The EGJ and greater gastric curvature are mobilized from a point 5 cm proximal to the pylorus to the angle of His and left crura. Dissection typically begins along the greater curve of the stomach at the level of the angularis to facilitate access to the lesser sac. The dissection is carried out proximally, requiring division of the gastroepiploic and short gastric vessels using vessel sealing or ultrasonic dissectors until the angle of His is completely mobilized. Attention is then turned to distal dissection, and the greater curvature is freed from its attachments to approximately 5 cm proximal to the pylorus. Once the greater curvature is completely dissected free of its attachments, the orogastric tube is swapped for a nontapered elastic Bougie (32–36F) to size the gastric sleeve. A laparoscopic stapler is then used to divide the stomach using sequential firings at an angle nearly parallel to the proximal lesser curve in close proximity to the Bougie, taking care to maintain nearly equal lengths of anterior and posterior stomach.^35^ Upon completion of stapling, integrity of the staple line is assessed and reinforced as needed. A leak test is performed with methylene blue solution and if no leaks are identified, the resected stomach and trocars are removed followed by laparoscopic port closure.

#### Endoscopic sleeve gastroplasty (ESG)

Indications for ESG include patients with BMI ≥ 30 Kg/m^2^ with or without obesity-related comorbidities (Figure 3B). Under general anesthesia, a dual channel gastroscope is introduced into the stomach and suture site marking is performed using 30W argon plasma coagulation on the anterior and posterior gastric walls. With the help of a helix tissue grasper, the stomach is imbricated with an average of 7 to 9 full-thickness permanent sutures in a running “U’ shaped plication, from the antral incisura to the gastroesophageal junction, with fundal sparing, to achieve gastric body tubularization.^34^ The “U” plications can be reinforced with an average of 3 to 4 superimposed sutures in a triangular pattern resulting in a shorter tubular and sleeve-like stomach. The patients get typically discharged same day or less likely admitted for overnight observation.

#### Comparison

Though ESG results in a remarkable 1-year total body weight loss (%TBWL) of 16.3%, LSG is superior with a %TBWL of 23.4%.^34^^,^^36^ LSG superiority was confirmed in a meta-analysis of 2188 patients resulting in significantly more excess weight loss compared to ESG (80.3% vs 62.2%, *P* = .001).^37^ Both procedures have a remarkable impact on obesity-related comorbidities.^34^^,^^38^^,^^39^ At 4–5 years follow up, LSG and ESG were found to offer durable weight loss with a %TBWL of 17.8% and 15.9%, respectively.^36^^,^^40^ For patients who regain weight after LSG, ESG provides an effective treatment option.^41^^,^^42^ In terms of safety and postprocedure recovery, ESG offers significantly lower procedure duration [64 min (95% confidence interval (CI): 58, 70) vs 81 min (95% CI: 80, 82)], hospital length of stay [0.5 day (95% CI: 0.3, 0.7) vs 1.4 days (95% CI: 1.41, 1.45)], adverse events (odds ratio = 0.33, 95% CI: 0.16, 0.68), and new-onset GERD (1.9% vs 14.5%, *P* < .05) compared to LSG.^43^, ^44^, ^45^

### Heller Myotomy → Peroral Endoscopic Myotomy (POEM)

**Figure 4:**
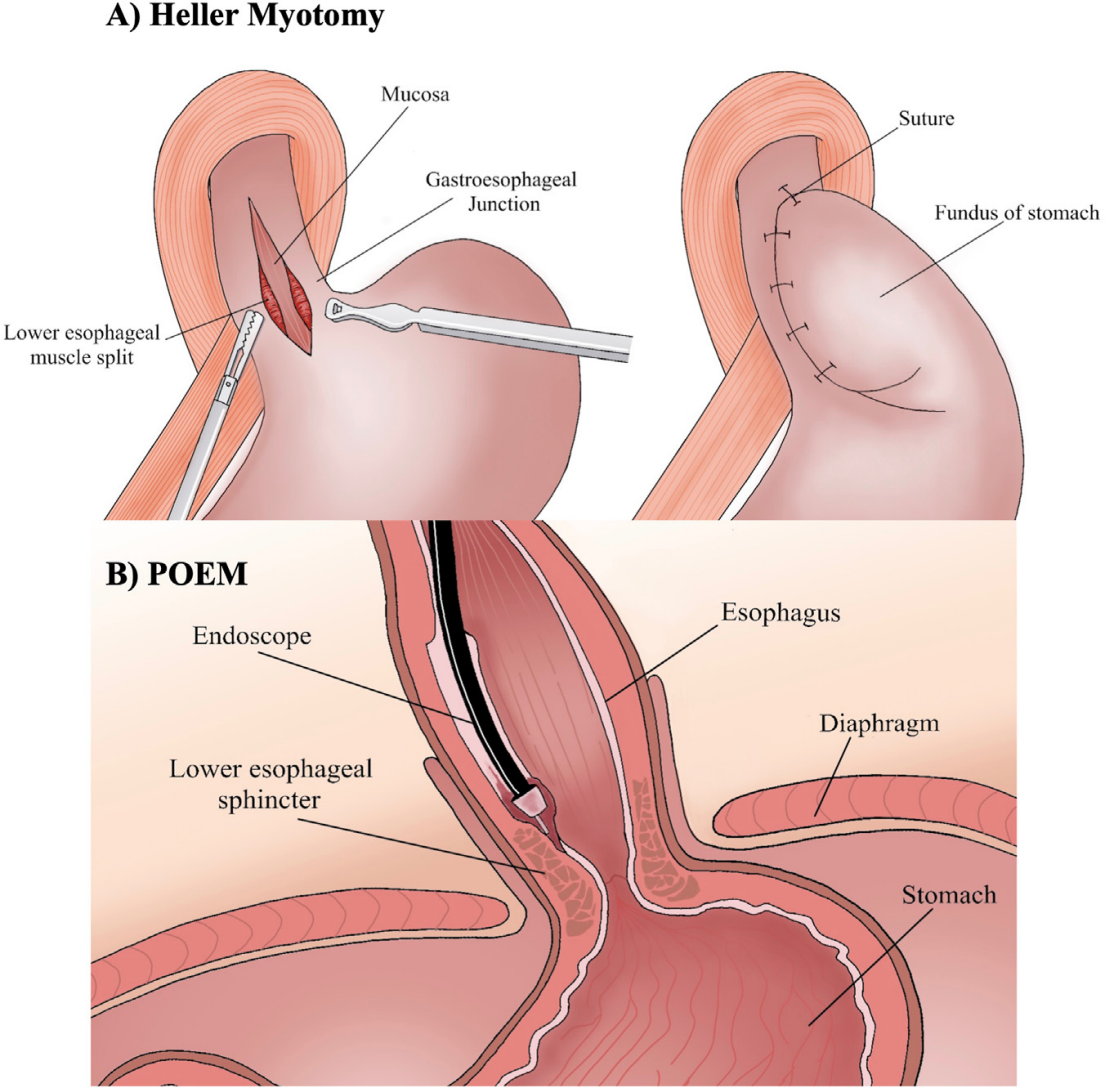
Illustration of Heller myotomy (A) vs peroral endoscopic myotomy (B).

Achalasia is an esophageal motility disorder characterized by impaired relaxation of the LES (Figure 4). While the exact pathophysiologic underpinnings of achalasia have yet to be elucidated, dysfunction of the LES is assumed to result from inflammation and degeneration of neurons in the distal esophageal wall. While specific antigenic agents have not been identified, association of achalasia with variants in the human leukocyte antigens genes and presence of antienteric neuron antibodies suggest achalasia may be an autoimmune disorder.^46^, ^47^, ^48^ Achalasia is commonly associated with dysphagia and weight loss. Concurrent esophageal spasm can be associated with chest pain. Because the underlying pathophysiologic insult that leads to loss of inhibitory nerve function in the smooth muscle of the esophagus has yet to be elucidated, medical and surgical management is centered on reduction of LES tone.

Over a century ago, Ernst Heller first introduced anterior and posterior myotomy of the distal esophagus and cardia as a method of reducing LES tone in patients with achalasia. During early iterations of the technique, the surgery was performed via both left thoracotomy and laparotomy. As with other surgical treatments for gastrointestinal disorders, there has been a shift toward minimally invasive approaches for achalasia. While the thoracoscopic approach yielded consistent and durable symptomatic improvement,^49^ it required exclusion of the left lung and chest tube placement which were both associated with additional respiratory complications.^50^^,^^51^ Additionally, 60% of patients undergoing thoracoscopic myotomy were found to have significant reflux on pH monitoring performed postoperatively.^50^ These shortcomings led to development of the surgical approach used today, the laparoscopic heller myotomy (LHM) with fundoplication.^51^ Peroral endoscopic myotomy (POEM), performed for the first time in Japan in 2008 by Inoue, avoids the risks of laparoscopy and thoracoscopy altogether and represents the next step in the evolution of minimally invasive management of achalasia.

#### Heller myotomy

During Heller myotomy, the patient is positioned in the supine position. Pneumoperitoneum is established and 3–4 trocars are placed under direct visualization (Figure 4A). The gastric fundus is dissected, and the short gastric vessels divided using a harmonic scalpel. The EGJ is exposed before myotomy is performed longitudinally along the anterior aspect of the esophageal wall.

Fundoplication is then performed to mitigate the risk of postmyotomy reflux. Given the absence of esophageal peristalsis in patients with achalasia, total fundoplication often results in recurrent dysphagia. Therefore, several techniques have been developed to combine partial fundoplication with myotomy. Dor developed a modified Heller-Nissen technique that involved using 2 rows of sutures to secure the anterior wall of the stomach to both sides of the myotomy. Conversely, Toupet proposed a performing posterior fundoplication, again with 2 rows sutures to attach the gastric fundus to either side of the myotomy.^52^

#### Peroral endoscopic myotomy (POEM)

In the earliest myotomies, endoscopists simply passed an endoscope into the esophagus and made 1 1-cm long incisions through the mucosa to cut the circular fibers above the Z-line (Figure 4B). This approach raised considerable risk of mediastinal contamination from esophageal content. Pasricha et al. introduced a novel technique in which a submucosal tunnel was created to directly visualize the muscular layer before myotomy.^53^ The mucosal flap could then be closed to prevent leakage of luminal contents. Inoue et al. introduced use of endoscopic submucosal dissection technique to create the submucosal tunnel, use of a triangle-tip knife to precisely dissect circular muscle layers and the use of positive pressure ventilation during the POEM to reduce the risk of mediastinal emphysema.^54^ This procedure remains the most performed endoscopic technique for the treatment of achalasia today. Much like with surgical myotomy, postprocedural GERD following POEM is common.

#### Comparison

In a multicenter randomized controlled trial (RCT), 221 patients with achalasia were randomized to either POEM or LHM.^55^ After 24 months of follow-up, 83% of patients in the POEM group and 81.7% of patients in the LHM group achieved clinical success (Eckardt symptom score of 3 or less), suggesting POEM was noninferior to LHM. Operative times and hospital stays were both significantly shorter in the POEM group. Another single-center RCT randomized patients to POEM or laparoscopic myotomy with partial fundoplication and evaluated treatment success, defined as at least a 3-point reduction in Eckardt score, LES pressure <15 mmHg, and a >50% reduction in height of barium column at 1 minute. There was no difference in symptomatic improvement at 1, 6, or 12 months of follow-up, LES pressure by manometry, adverse events, or length of stay. POEM resulted in improvements in all domains of the quality of life questionnaire whereas the laparoscopic myotomy with partial fundoplication group experienced improvements in only 3 of 8 total domains. Importantly, reflux esophagitis was a more common following POEM.^56^ In pooled meta-analysis comparing POEM and Heller myotomy with fundoplication, abnormal acid exposure was seen in 39% and 17% of patients across 17 and 28 studies, respectively.^57^ The optimal myotomy length from distal esophagus into the cardia remains a topic of debate given its role in the incidence of post-POEM GERD.^58^ The use of endoluminal functional lumen imaging probes (EndoFLIP, Medtronic, Dublin, Ireland) for real-time measurement of distensibility has allowed endoscopists to tailor myotomy length and depth.^59^ The optimal distensibility index on EndoFLIP is a topic of current research. Nonetheless, given the prevalence of post-POEM GERD, attempts to combine POEM with concomitant fundoplication are also under development.^60^, ^61^, ^62^

### Zenker Open Diverticulectomy → Endoscopic Diverticulotomy

Zenker’s diverticulum (ZD) refers to outpouching of the esophagus between the cricopharyngeal (CP) muscle and the inferior pharyngeal constrictor muscle. Abnormal function of the CP muscle or congenital weakness is hypothesized to be central to development of the diverticulum. Both increased tone of the CP muscle and eventual extrinsic compression from the food-filled diverticulum likely contribute to symptoms of dysphagia and regurgitation. Some of the earliest surgical treatments involved the creation of a fistula from skin to diverticulum for drainage. Others attempted simple inversion of the diverticular sac into the esophagus. Both these early methods failed to produce reliable or durable results.^63^ Myotomy was later implemented, becoming a central surgical treatment today which can involve diverticular sac affixation to the prevertebral fascia (diverticulopexy) or inversion into the esophageal lumen (invagination). Harris Mosher made the first attempt to manage ZD endoscopically by dividing the common wall between the diverticulum and esophagus using surgical scissors passed through the rigid endoscope.^64^ Given the high mortality rate with initial cases, the procedure did not gain popularity. In 1960, Dohlman modified the procedure using an electrocautery device and reported no deaths or severe complications across 100 patients.^65^ With advancements of third space endoscopy, ZD peroral endoscopic myotomy (Z-POEM) gained popularity as a minimally invasive approach.

#### Surgical Zenker’s diverticulectomy

Today, open surgical repair of ZD is performed under general anesthesia, though local anesthetic or selective spinal anesthesia may also be utilized. After positioning of the patient with the neck in hyperextension, an incision is made just ventral to the sternocleidomastoid muscle. Subcutaneous tissue, overlying muscle, and fascia are divided to expose the pharynx and cervical esophagus. The diverticular pouch is identified and extensively dissected from the surrounding connective tissue. During dissection, care must be taken to avoid several of many delicate structures in the area, including the recurrent laryngeal nerve, external laryngeal nerve, hypoglossal nerve, and cervical cutaneous nerve. Myotomy of the CP muscle is performed. The diverticular sac is the excised. A drain is typically placed and removed after 24–48 hours.^66^

#### Zenker’s diverticulum (ZD) peroral endoscopic myotomy (Z-POEM)

While flexible endoscopic diverticulotomy can be performed to mimic the aforementioned technique surgeons use with rigid endoscopes, Z-POEM technique leverages endoscopic submucosal dissection to perform a more precise and complete myotomy. During Z-POEM, the septum is identified endoscopically, and a mucosal incision is made just proximally. Dissection is performed to create a submucosal tunnel leading to the muscular septum. The tunnel is extended on both the diverticular and esophageal side. Injection of indigo carmine-mixed saline can be used to expand the potential spaces on either side of the longitudinal muscle fibers. After adequate exposure is achieved, complete septotomy is performed. The mucosectomy site is then closed using clips.

#### Comparison

While head-to-head data for surgical and endoscopic modalities of ZD treatment are scarce, Z-POEM offers several theoretical advantages over alternate approaches. With direct visualization of the muscular septum, endoscopists can perform complete myotomy compared to incomplete myotomy surgically to avoid leakage and mediastinitis. Z-POEM has demonstrated clinical success rates >90% with perforation rates near 5% in a large, international, multicenter study.^67^ A meta-analysis of 11 studies evaluating Z-POEM demonstrated similar success rates with a pooled recurrence rate of 11.2%.^68^ Compared to surgical or rigid endoscopic approaches, Z-POEM offers various advantages including favorable efficacy, lower morbidity & mortality, better tolerance, and potentially lower recurrence rates.^69^^,^^70^

### Videoscopic-Assisted Retroperitoneal Debridement → EUS Guided Cystogastrostomy

**Figure 5:**
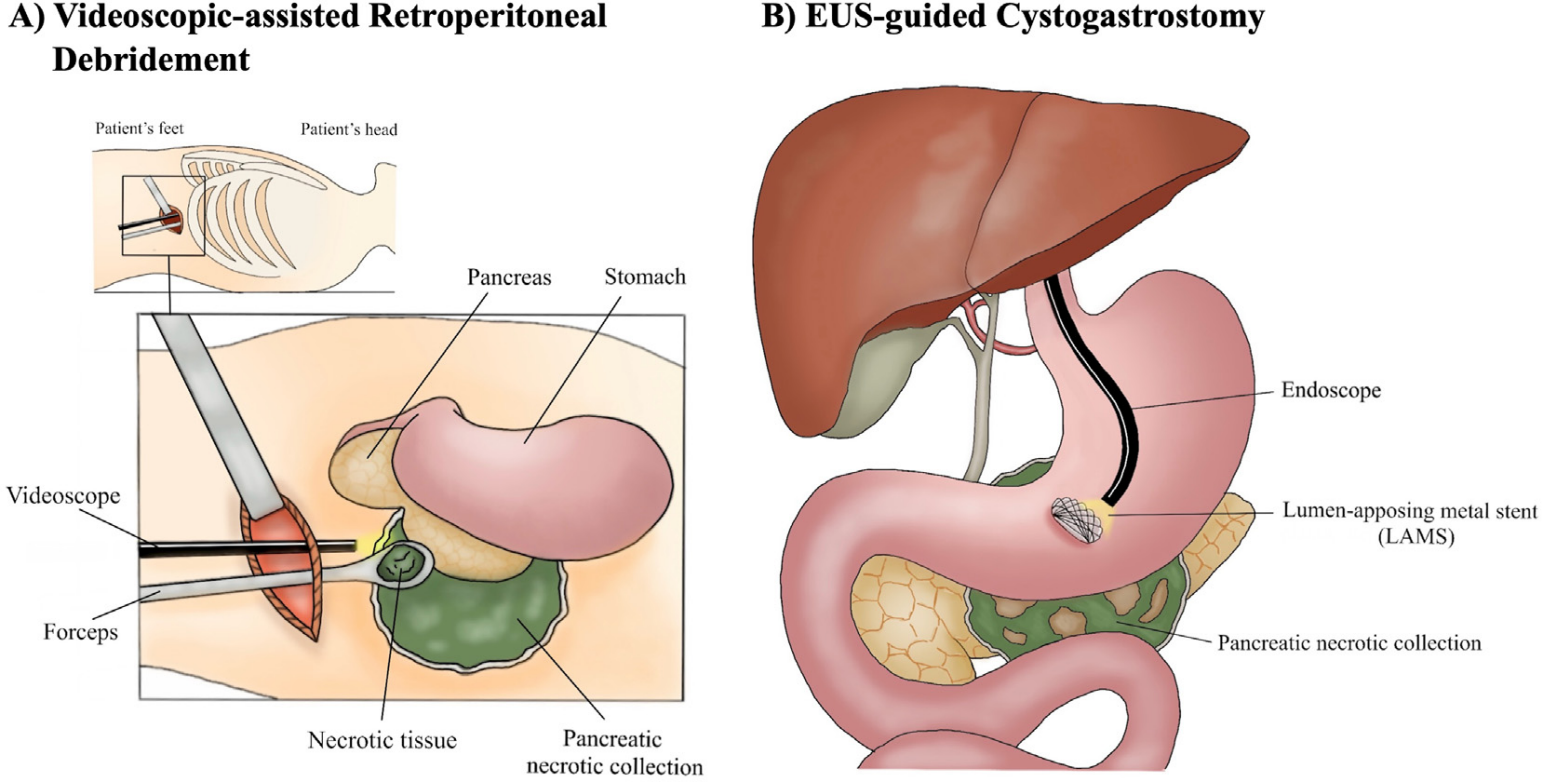
Illustration of videoscopic assisted retroperitoneal debridement (A) vs EUS guided cystogastrostomy (B).

Acute pancreatitis remains one of the most common gastrointestinal causes of hospitalization in the US (Figure 5).^71^ while most cases are considered mild and self-limiting in the early phase (<1 week), some patients develop necrotizing pancreatitis in the late phase (>1 week) leading to sepsis and organ failure.^72^ Pancreatic fluid collections (PFCs) are a local complication of acute pancreatitis that can be categorized based on the revised Atlanta Classification. Acute PFCs (< 4 weeks) include acute peripancreatic fluid collections and acute necrotic collections. Chronic PFCs (≥4weeks) with a well-defined wall include pseudocyst and wall-offed necrosis (WON).^73^

The drainage of PFCs is indicated in patients with infected necrosis, preferably at the stage of WON. Infected necrosis diagnosis is supported clinically and by gas presence on CT scan.^72^ Other indications for drainage include large collections complicated by bowel or biliary obstruction, persistent symptoms, recurrent acute pancreatitis, fistula, abdominal compartment syndrome, or persistent systemic inflammatory response syndrome.^72^^,^^74^^,^^75^ Drainage can be performed surgically, percutaneously, or endoscopically. Based on the American Gastroenterological Association Clinical Practice Update in 2020, percutaneous drainage (PCD) and endoscopic transmural drainage (ETD) are the first line for managing WON as minimally invasive approaches compared to surgery.^75^

#### Surgical approaches

Surgical approaches for WON include video-assisted retroperitoneal debridement (VARD), laparoscopic cystogastrostomy, and laparoscopic or open debridement (Figure 5A). VARD is considered a minimally invasive surgery as it consists of an image-guided percutaneous catheter placement into the retroperitoneum via the left flank, followed by an intracavitary videoscopic necrosectomy. Surgical debridement via laparoscopic or open technique by accessing the lesser sac via the greater omentum, through the transverse mesocolon, or the lesser omentum.^75^

Laparoscopic cystogastrostomy requires 4 ports (camera port at the umbilicus, 2 working ports in bilateral pararectus position, and fourth in the epigastric region for puncturing and aspiration of pancreas fluid collection).^76^ Access to the posterior gastric wall is established through an anterior gastrostomy with the help of electrocautery. Laparoscopic ultrasound, if available, is used to localize the cyst and to identify collaterals or any other vascular structures. A long Veress needle is used to confirm the location of the pseudocyst by aspiration and to sample the fluid. A 15 cm long, 12 mm port is then introduced through the posterior wall of the stomach into the cyst using the Veress needle as a guide, and cyst contents aspirated. The cyst cavity is then irrigated with normal saline via the long port. Cystogastrostomy is then performed using an endostapler. Debris is gently removed from the cyst cavity using blunt dissection. Once hemostasis is achieved, the gastrotomy is closed in 2 layers.

#### Endoscopic drainage of pancreatic fluid collection

ETD is performed by introducing a needle into the PFC via gastric or duodenal walls. Then, a guidewire is inserted followed by a stent to create a fistula for drainage (Figure 5B). Pancreatic head collections are usually drained transduodenally, while the others are drained transgastrically.^77^ Despite the lack of study comparing ETD with and without endoscopic ultrasound (EUS), several experts recommend using EUS to increase technical success and prevent iatrogenic injury to adjacent vessels or organs.^75^^,^^78^ There are 3 stents used in ETD including the plastic pigtail stent, self-expandable metallic stent (SEMS), and lumen apposing metal stents (LAMS). It is more commonly used as it was designed to prevent stent migration and leakage.^79^ Removal of necrotic tissue known as endoscopic transluminal necrosectomy can also be performed using LAMS. A multicenter retrospective study in the U.S. report the superiority of LAMS compared to plastic stents in WON including higher clinical success, shorter procedure time, lower rates of surgery and recurrence.^80^ These findings are consistent with previously published literature.^81^^,^^82^ On the contrary, a meta-analysis of 30 studies showed the similar risk of bleeding and perforation between LAMS and plastic stent.^83^ Therefore, the decision to use either plastic stent or LAMS is based on the endoscopist’s clinical judgment and experience. In addition to stents placement, nasocystic drain has also been used for PFC with viscous debris offering higher short-term clinical success and lower stent occlusion, but patient’s discomfort can be a limiting factor.^84^

#### Comparison

The landmark Step Up trial radically changed the management of WON.^85^ Whereas open necrosectomy was previously the standard of care, this has fallen in favor of minimally invasive surgery due to a lower risk of mortality.^85^^,^^86^ This has been followed by a paradigm shift to endoscopic approaches. Compared to VARD, a multicentric RCT from Netherland showed significantly lower rates of major complications and inflammatory response with ETD.^87^ Similarly, a subsequent RCT showed a significantly reduced major complications, lower costs, and increased quality of life of patients with infected necrotizing pancreatitis who underwent ETD compared to VARD or laparoscopic debridement.^88^ However, the TENSION RCT did not demonstrate a difference in mortality and morbidity between endoscopic (ETD and endoscopic necrosectomy as needed) and surgical step-up approach (PCD and VARD as needed) groups.^73^ However, the ETD group tended to have a shorter hospital stay (*P* = .014) and less pancreatic fistulas (*P* = .001).

Data comparing ETD to laparoscopic cystogastrostomy is limited to few small RCTs. One RCT revealed similar efficacy but higher postprocedural infection in ETD group (*P* = .01).^89^ Another RCT revealed similar efficacy, major complications, and reinterventions between EDT and laparoscopic cystogastrostomy, but significantly shorter hospital stay and lower costs with ETD.^90^ Overall, ETD appears to be a preferred option over surgical approaches as it provides better clinical outcomes in patients with PFCs. When endoscopy is not feasible or PFCs are difficult to access via endoscopy, minimally invasive surgery or PCD should be considered as alternative options. A multidisciplinary approach involving gastroenterologists, intervention radiologists, and surgeons is required to achieve technical and clinical success.

### Surgical Gastrojejunostomy → EUS Guided GJ

**Figure 6:**
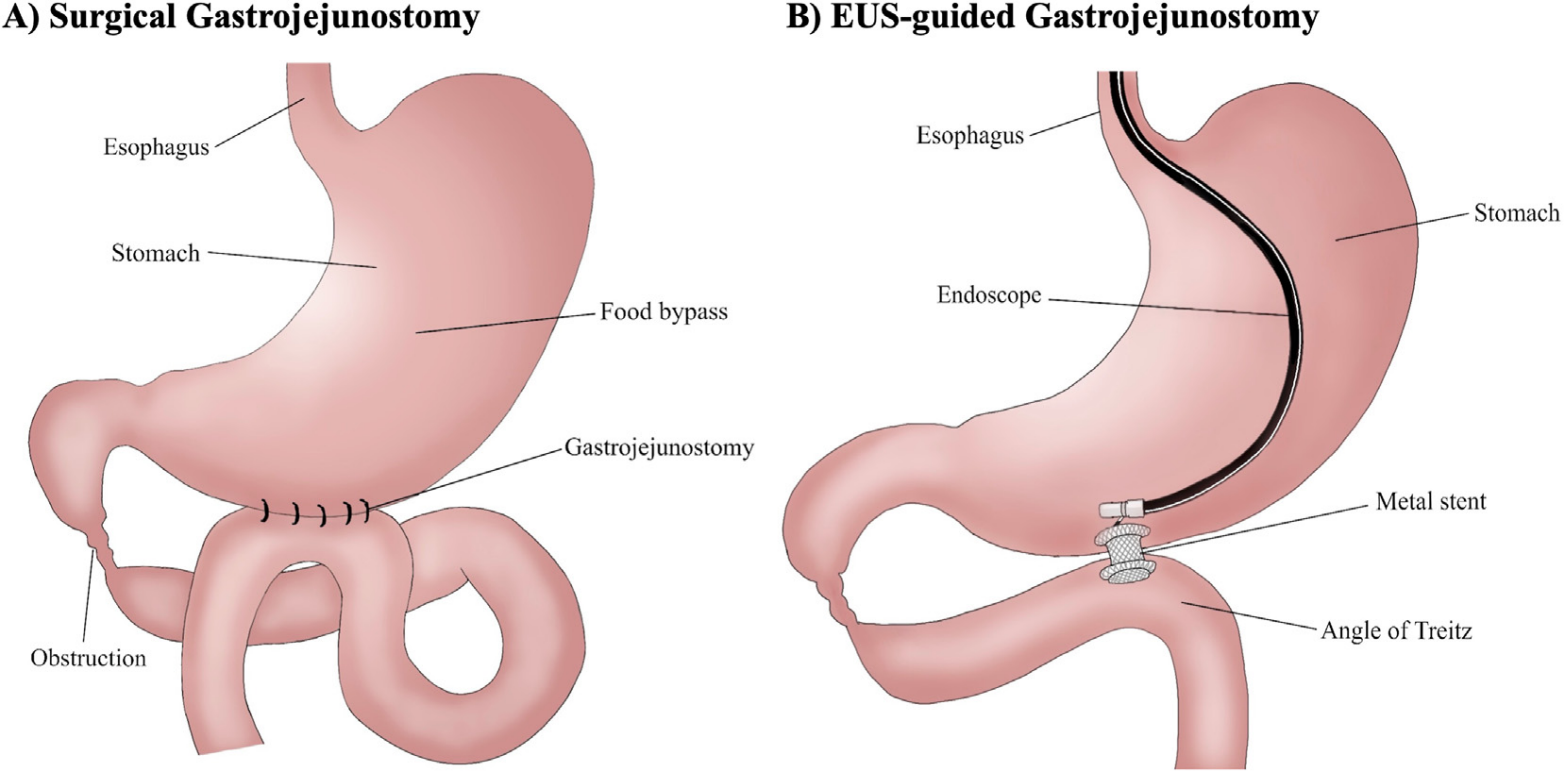
Illustration of surgical gastrojejunostomy (A) vs EUS-guided gastrojejunostomy (B).

Gastric outlet obstruction (GOO) is a gastrointestinal condition resulting from mechanical obstruction of distal stomach, pylorus, or duodenum (Figure 6). While malignant etiologies (gastroduodenal, pancreaticobiliary, and others) are predominant, peptic ulcer disease is a common benign cause.^91^ Since the 19^th^ century, surgical Gastrojejunostomy (SGJ) has been implemented in the GOO treatment, first being by Anton Woefler treating a pyloric neoplasm.^92^^,^^93^ Dr Theodor Billroth later performed Billroth 2 procedure to anastomose the stomach to the jejunum without a separate limb for pancreaticobiliary secretions.^92^ The Roux-en-Y procedure was another variation performed by Anton Woefler in 1883 that was later popularized by Cesar Roux in which a gastrojejunostomy anastomosis is created between the stomach and jejunal loop. In the 1990s, endoscopic placement of SEMS across the obstruction presented a minimally invasive alternative to SGJ.^94^^,^^95^ Over the last 10 years, endoscopic ultrasound-guided gastrojejunostomy (EUS-GJ) using LAMS became a popular option for GOO.

#### Original surgical concept: (surgical gastrojejunostomy)

Following induction of general anesthesia, the patient is prepped and draped in the usual sterile fashion and placed in supine position with arms abducted at right angles (Figure 6A).^93^ A nasogastric or orogastric tube is placed to decompress the stomach. A midline epigastric incision is made and extended upward to the xiphoid process and downward to the umbilicus. The stomach and duodenum are exposed and the lesser sac is entered through the greater omentum at the level of the gastroepiploic arcade. A loop of jejunum 40 cm from the ligament of Trietz is identified and approximated to the back wall of the body of the stomach in an antecolic fashion.^92^ Then, the surgical anastomosis can either be sewn by hand or performed using a linear stapling device to create a side-to-side stapled anastomosis, which is subsequently interrogated for leaks. This procedure is also commonly performed using a laparoscopic technique. The trocars used include a 5-mm port to the left of the umbilicus for the camera, a 5-mm right midabdominal trocar which is later exchanged for a 12-mm port to accommodate the linear stapler needed to perform the anastomosis, and a 5-mm left subcostal trocar for the assistant working port.

#### Current endoscopic approach: (endoscopic ultrasound guided gastrojejunostomy)

An endoscope was advanced into the stomach and to the area of obstruction (Figure 6B). 500cc of water mixed with methylene blue, and contrast are injected into the small intestine using a 10Fr naso-biliary tube. Next, the echoendoscope is advanced into the stomach facing a dilated loop of small intestine. Using a 19G needle, the small intestine is punctured, and methylene blue tinted fluid is aspirated. Following, a 15-mm or 20 mm × 10 mm cautery-assisted LAMS is directly advanced into the jejunum limb across the stomach wall under direct endosonographic guidance. Fluoroscopic assistance can be used. Finally, a 7Fr x 5-cm plastic double pigtail stent is deployed across the LAMS for anchoring purposes.

#### Comparisons

A meta-analysis by Kirshnamoorthi et. al., compared SGJ, SEMS and EUS-GJ and showed that technical success was lower for EUS-GJ (95.3%) compared to surgical GJ (99.9%).^96^ However, the clinical success rates of the 3 procedures were comparable (92.3% vs 88.9% vs 89%; respectively), with clinical success defined as relief of obstructive symptoms and improved oral intake. In terms of recurrent GOO, EUS-GJ showed the lowest rate (4%) compared to SEMS (28.7%) and SGJ (16.9%). Similarly, reintervention was also lower with EUS-GJ (11.2%) compared to duodenal SEMS (20.3%) and SGJ (12.6%). Periprocedural bleeding (1.7% vs 2.9% vs 5.2%) and perforation rates (1.6% vs 2.8% vs 2%) were comparable between SEMS, EUS-GJ, and SGJ, respectively.^96^

Although EUS-GJ appeals as a favorable alternative to SGJ given its shorter hospital stay and lower cost, there are important factors that determine eligibility for EUG-GJ including life expectancy, tumor location, and ascites presence.^96^^,^^97^ Shorter survival (<6 months) and poor surgical candidacy favors EUS-GJ over SGJ in malignant GOO.^95^

## Discussion

The evolution of endoscopic techniques and emphasis on multidisciplinary care have led to the development of a wide array of minimally invasive approaches to multiple gastrointestinal conditions highlighted in this article (Table). These tools are valuable for many patients whose comorbidities have limited their surgical candidacy. However, the multidisciplinary discussion and collaboration between endoscopists and surgeons remain pivotal to identify the optimal patient-centered therapy on a case-by-case basis. Hence, we should continue to invest in growing our integrative relationships and programs to deliver ideal care to our patients. In this article, we discuss the cutting-edge endoscopic interventions and their origins in established surgical techniques.

While neither surgical nor endoscopic techniques dominate the management of most of the conditions described, with the notable exception of PEG tube placement, future technological advances and clinical studies may tilt the balance toward endoscopic therapy. Given the arrival of EndoFLIP to treat post-POEM GERD for example, a future in which POEM overtakes Heller myotomy for the treatment of achalasia is easy to envision. More likely however, is the emergence of collaborative approaches like the combined TIF procedure during which the hiatal hernia is fixed surgically followed by the recreation of the gastroesophageal flap valve endoscopically for the treatment of GERD.^17^

Nuanced consideration of the benefits and disadvantages of each therapeutic approach should be undertaken on a case-by-case basis. For example, while ESG is a less invasive intervention and carries lower risk of postprocedural GERD, LSG offers more TBWL% than ESG and is more widely available. Furthermore, EUS-guided cystogastrostomy is noninferior to VARD and offers significantly lower major complication rates but requires more total interventions for complete debridement which can be difficult to coordinate in nonadherent patients or those with limited access to care. Finally, EUS-GJ as a treatment for GOO presents a significantly less invasive and more favorable short-term palliative approach than surgical GJ but comes at the cost of lower durability and stent-related dietary restrictions.

### A Look Toward the Future

If one had a crystal ball to predict the future of endoscopy today, it would likely show an incorporation of multiple currently used “trendy procedures” into mainstream clinical practice. Two critical factors which could impact this progression come to mind. First, with the near dominance of laparoscopic surgery, followed by the adaptation of robotic surgery in the past 2 decades, significant changes are happening in the surgical arena. It is likely that our endoscopic approach will embrace such changes and likely follow the concept of telepresence, allowing for more meticulous, delicate, and safer interventions. Second, artificial intelligence (AI) is well-suited to leverage endoscopic data to improve endoscopy-associated outcomes. Indeed, endoscopy represents a prime field for the application of AI application due focus on visual identification, technology and devices, and data-driven clinical decision-making. Many studies have focused on utilizing AI application in GI; however, the practical application of this is considered preliminary as of this writing. Third, our routine diagnostic procedures in today's practice may not be as dominant in the future. For example, screening colonoscopy, a procedure that has decreased colorectal cancer prevalence and mortality, backed by a large body of literature, could be replaced by a simple stool or even a blood test. Similarly, tumor markers can potentially become the standard method for various detection and screening protocols. Overall, the future is auspicious for endoscopists and patients alike as less invasive endoscopic techniques continue to be developed for many conditions. Expectedly, this will also be accompanied by broader expertise availability and collaboration between surgeons and endoscopists.

## Conclusion

The field of interventional and therapeutic endoscopy has matured markedly to the point of almost separating itself as a standalone specialty close to and simultaneously distinct from both general gastroenterology and minimal invasive surgery. As our arsenal of tools continues to expand, more high quality clinical studies will be needed to guide optimal treatment.

## References

[1] Ponsky J.L. (2011). The development of PEG: How it was. J Interv Gastroenterol.

[2] Gauderer M.W., Ponsky J.L., Izant R.J. (1980). Gastrostomy without laparotomy: a percutaneous endoscopic technique. J Pediatr Surg.

[3] Kandil E., Alabbas H., Jacob C. (2010). A simple and safe minimally invasive technique for laparoscopic gastrostomy. JSLS.

[4] Villalona G.A., McKee M.A., Diefenbach K.A. (2011). Modified laparoscopic gastrostomy technique reduces gastrostomy tract dehiscence. J Laparoendosc Adv Surg Tech A.

[5] Hsieh J.S., Wu C.F., Chen F.M. (2007). Laparoscopic Witzel gastrostomy--a reappraised technique. Surg Endosc.

[6] Rahnemai-Azar A.A., Rahnemaiazar A.A., Naghshizadian R. (2014). Percutaneous endoscopic gastrostomy: indications, technique, complications and management. World J Gastroenterol.

[7] Bankhead R.R., Fisher C.A., Rolandelli R.H. (2005). Gastrostomy tube placement outcomes: comparison of surgical, endoscopic, and laparoscopic methods. Nutr Clin Pract.

[8] Kohli D.R., Kennedy K.F., Desai M. (2021). Safety of endoscopic gastrostomy tube placement compared with radiologic or surgical gastrostomy: nationwide inpatient assessment. Gastrointest Endosc.

[9] Zachariah R.A., Goo T., Lee R.H. (2020). Mechanism and pathophysiology of gastroesophageal reflux disease. Gastrointest Endosc Clin N Am.

[10] Allison P.R. (1951). Reflux esophagitis, sliding hiatal hernia, and the anatomy of repair. Surg Gynecol Obstet.

[11] Mackay E.M., Louie B. (2023). Evolution in the treatment of gastroesophageal reflux disease over the last century: from a crural-centered to a lower esophageal sphincter–centered approach and back. Dis Esophagus.

[12] Nissen R. (1937). Die transpleurale resektion der Kardia. Dtsch Zschr Chir.

[13] Nissen R. (1961). Gastropexy and “fundoplication” in surgical treatment of hiatal hernia. Am J Dig Dis.

[14] Ellison E.C., Zollinger R.M. (2016). Zollinger's Atlas of Surgical Operations.

[15] Seeras K., Bittar K., Siccardi M.A. (2023). StatPearls [Internet].

[16] Chang K.J., Bell R. (2020). Transoral incisionless fundoplication. Gastrointest Endosc Clin N Am.

[17] Choi A.Y., Roccato M.K., Samarasena J.B. (2021). Novel interdisciplinary approach to GERD: concomitant laparoscopic hiatal hernia repair with transoral incisionless fundoplication. J Am Coll Surg.

[18] Bell R.C., Mavrelis P.G., Barnes W.E. (2012). A prospective multicenter registry of patients with chronic gastroesophageal reflux disease receiving transoral incisionless fundoplication. J Am Coll Surg.

[19] Bell R.C., Barnes W.E., Carter B.J. (2014). Transoral incisionless fundoplication: 2-year results from the prospective multicenter U.S. study. Am Surg.

[20] Hakansson B., Montgomery M., Cadiere G.B. (2015). Randomised clinical trial: transoral incisionless fundoplication vs. sham intervention to control chronic GERD. Aliment Pharmacol Ther.

[21] Hunter J.G., Kahrilas P.J., Bell R.C. (2015). Efficacy of transoral fundoplication vs omeprazole for treatment of regurgitation in a randomized controlled trial. Gastroenterology.

[22] Trad K.S., Fox M.A., Simoni G. (2017). Transoral fundoplication offers durable symptom control for chronic GERD: 3-year report from the TEMPO randomized trial with a crossover arm. Surg Endosc.

[23] Frazzoni M., Conigliaro R., Manta R. (2011). Reflux parameters as modified by EsophyX or laparoscopic fundoplication in refractory GERD. Aliment Pharmacol Ther.

[24] Richter J.E., Kumar A., Lipka S. (2018). Efficacy of laparoscopic Nissen fundoplication vs transoral incisionless fundoplication or proton pump inhibitors in patients with gastroesophageal reflux disease: a systematic review and network meta-analysis. Gastroenterology.

[25] Abu Dayyeh B., Murad M.H., Bazerbachi F. (2018). Efficacy of laparoscopic Nissen fundoplication vs transoral incisionless fundoplication or proton pump inhibitors in patients with gastroesophageal reflux disease: misleading ranking probabilities in network meta-analysis. Gastroenterology.

[26] Majeed M., Majeed S., Nagabhushanam K. (2021). Lesser investigated natural ingredients for the management of obesity. Nutrients.

[27] Rosenthal R.J., International Sleeve Gastrectomy Expert Panel, Diaz AA, et al (2012). International sleeve gastrectomy expert panel consensus statement: best practice guidelines based on experience of >12,000 cases. Surg Obes Relat Dis.

[28] Acosta A., Camilleri M., Shin A. (2015). Quantitative gastrointestinal and psychological traits associated with obesity and response to weight-loss therapy. Gastroenterology.

[29] Hess D.S., Hess D.W. (1998). Biliopancreatic diversion with a duodenal switch. Obes Surg.

[30] Eisendrath P., Cremer M., Himpens J. (2007). Endotherapy including temporary stenting of fistulas of the upper gastrointestinal tract after laparoscopic bariatric surgery. Endoscopy.

[31] Eubanks S., Edwards C.A., Fearing N.M. (2008). Use of endoscopic stents to treat anastomotic complications after bariatric surgery. J Am Coll Surg.

[32] Thompson C.C., Slattery J., Bundga M.E. (2006). Peroral endoscopic reduction of dilated gastrojejunal anastomosis after Roux-en-Y gastric bypass: a possible new option for patients with weight regain. Surg Endosc.

[33] Brethauer S.A., Chand B., Schauer P.R. (2012). Transoral gastric volume reduction as intervention for weight management: 12-month follow-up of TRIM trial. Surg Obes Relat Dis.

[34] Abu Dayyeh B.K., Bazerbachi F., Vargas E.J. (2022). Endoscopic sleeve gastroplasty for treatment of class 1 and 2 obesity (MERIT): a prospective, multicentre, randomised trial. Lancet.

[35] Ramos A.C., Bastos E.L., Ramos M.G. (2015). Technical aspects of laparoscopic sleeve gastrectomy. Arq Bras Cir Dig.

[36] Maciejewski M.L., Arterburn D.E., Van Scoyoc L. (2016). Bariatric surgery and long-term durability of weight loss. JAMA Surg.

[37] Marincola G., Gallo C., Hassan C. (2021). Laparoscopic sleeve gastrectomy versus endoscopic sleeve gastroplasty: a systematic review and meta-analysis. Endosc Int Open.

[38] Trastulli S., Desiderio J., Guarino S. (2013). Laparoscopic sleeve gastrectomy compared with other bariatric surgical procedures: a systematic review of randomized trials. Surg Obes Relat Dis.

[39] Hajifathalian K., Mehta A., Ang B. (2021). Improvement in insulin resistance and estimated hepatic steatosis and fibrosis after endoscopic sleeve gastroplasty. Gastrointest Endosc.

[40] Sharaiha R.Z., Hajifathalian K., Kumar R. (2021). Five-year outcomes of endoscopic sleeve gastroplasty for the treatment of obesity. Clin Gastroenterol Hepatol.

[41] de Moura D.T.H., Barrichello S., de Moura E.G.H. (2020). Endoscopic sleeve gastroplasty in the management of weight regain after sleeve gastrectomy. Endoscopy.

[42] Maselli D.B., Alqahtani A.R., Abu Dayyeh B.K. (2021). Revisional endoscopic sleeve gastroplasty of laparoscopic sleeve gastrectomy: an international, multicenter study. Gastrointest Endosc.

[43] Fayad L., Schweitzer M., Itani M. (2022). Does endoscopic mean safer? A comparison of the short-term safety of endoscopic versus laparoscopic bariatric therapies. Endosc Int Open.

[44] Fayad L., Adam A., Schweitzer M. (2019). Endoscopic sleeve gastroplasty versus laparoscopic sleeve gastrectomy: a case-matched study. Gastrointest Endosc.

[45] Phan J I.D. (2020).

[46] Wong R.K., Maydonovitch C.L., Metz S.J. (1989). Significant DQw1 association in achalasia. Dig Dis Sci.

[47] Verne G.N., Sallustio J.E., Eaker E.Y. (1997). Anti-myenteric neuronal antibodies in patients with achalasia. A prospective study. Dig Dis Sci.

[48] Verne G.N., Hahn A.B., Pineau B.C. (1999). Association of HLA-DR and -DQ alleles with idiopathic achalasia. Gastroenterology.

[49] Pellegrini C., Wetter L.A., Patti M. (1992). Thoracoscopic esophagomyotomy. Initial experience with a new approach for the treatment of achalasia. Ann Surg.

[50] Patti M.G., Arcerito M., De Pinto M. (1998). Comparison of thoracoscopic and laparoscopic Heller myotomy for achalasia. J Gastrointest Surg.

[51] Patti M.G., Pellegrini C.A., Horgan S. (1999). Minimally invasive surgery for achalasia: an 8-year experience with 168 patients. Ann Surg.

[52] Toupet A. (1963). [Technic of esophago-gastroplasty with phrenogastropexy used in radical treatment of hiatal hernias as a supplement to Heller's operation in cardiospasms]. Mem Acad Chir.

[53] Pasricha P.J., Hawari R., Ahmed I. (2007). Submucosal endoscopic esophageal myotomy: a novel experimental approach for the treatment of achalasia. Endoscopy.

[54] Inoue H., Minami H., Kobayashi Y. (2010). Peroral endoscopic myotomy (POEM) for esophageal achalasia. Endoscopy.

[55] Werner Y.B., Hakanson B., Martinek J. (2019). Endoscopic or surgical myotomy in patients with idiopathic achalasia. N Engl J Med.

[56] de Moura E.T.H., Jukemura J., Ribeiro I.B. (2022). Peroral endoscopic myotomy vs laparoscopic myotomy and partial fundoplication for esophageal achalasia: a single-center randomized controlled trial. World J Gastroenterol.

[57] Repici A., Fuccio L., Maselli R. (2018). GERD after per-oral endoscopic myotomy as compared with Heller’s myotomy with fundoplication: a systematic review with meta-analysis. Gastrointest Endosc.

[58] Hasan A., Low E.E., Fehmi S.A. (2022). Evolution and evidence-based adaptations in techniques for peroral endoscopic myotomy for achalasia. Gastrointest Endosc.

[59] Yoo I.K., Choi S.A., Kim W.H. (2019). Assessment of clinical outcomes after peroral endoscopic myotomy via esophageal distensibility measurements with the endoluminal functional lumen imaging probe. Gut Liver.

[60] Brewer Gutierrez O.I., Chang K.J., Benias P.C. (2022). Is transoral incisionless fundoplication (TIF) an answer to post-peroral endoscopic myotomy gastroesophageal reflux? A multicenter retrospective study. Endoscopy.

[61] Reddy N.D. (2019). Peroral endoscopic myotomy with fundoplication: are we there yet!. Endoscopy.

[62] Issa D., Benias P.C., Carr-Locke D.L. (2021). Achalasia and gastroparesis: Coexisting entities or consequence of therapy?. Endosc Int Open.

[63] Watemberg S., Landau O., Avrahami R. (1996). Zenker's diverticulum: reappraisal. Am J Gastroenterol.

[64] Constantin A., Mates I.N., Predescu D. (2012). Principles of surgical treatment of Zenker diverticulum. J Med Life.

[65] Dohlman G., Mattsson O. (1960). The endoscopic operation for hypopharyngeal diverticula: a roentgencinematographic study. AMA Arch Otolaryngol.

[66] Simic A., Radovanovic N., Stojakov D. (2009). Surgical experience of the national institution in the treatment of Zenker’s diverticula. Acta Chir Iugosl.

[67] Yang J., Novak S., Ujiki M. (2020). An international study on the use of peroral endoscopic myotomy in the management of Zenker’s diverticulum. Gastrointest Endosc.

[68] Zhang H., Huang S., Xia H. (2022). The role of peroral endoscopic myotomy for Zenker’s diverticulum: a systematic review and meta-analysis. Surg Endosc.

[69] Yuan Y., Zhao Y.F., Hu Y. (2013). Surgical treatment of Zenker’s diverticulum. Dig Surg.

[70] Ishaq S., Hassan C., Antonello A. (2016). Flexible endoscopic treatment for Zenker’s diverticulum: a systematic review and meta-analysis. Gastrointest Endosc.

[71] Mederos M.A., Reber H.A., Girgis M.D. (2021). Acute pancreatitis: a review. JAMA.

[72] Trikudanathan G., Wolbrink D.R.J., van Santvoort H.C. (2019). Current concepts in severe acute and necrotizing pancreatitis: an evidence-based approach. Gastroenterology.

[73] van Brunschot S., van Grinsven J., van Santvoort H.C. (2018). Endoscopic or surgical step-up approach for infected necrotising pancreatitis: a multicentre randomised trial. Lancet.

[74] Freeman M.L., Werner J., van Santvoort H.C. (2012). Interventions for necrotizing pancreatitis: summary of a multidisciplinary consensus conference. Pancreas.

[75] Baron T.H., DiMaio C.J., Wang A.Y. (2020). American gastroenterological association clinical practice update: management of pancreatic necrosis. Gastroenterology.

[76] Bansal V.K., Krishna A., Prajapati O.P. (2020). Outcomes following laparoscopic internal drainage of walled off necrosis of pancreas: experience of 134 cases from a tertiary care centre. Surg Endosc.

[77] Nabi Z., Basha J., Reddy D.N. (2017). Endoscopic management of pancreatic fluid collections-revisited. World J Gastroenterol.

[78] Varadarajulu S. (2008). EUS versus surgical cyst-gastrostomy for management of pancreatic pseudocysts. Gastrointest Endosc.

[79] Mussetto A., Fugazza A., Fuccio L. (2018). Current uses and outcomes of lumen-apposing metal stents. Ann Gastroenterol.

[80] Chen Y.I., Yang J., Friedland S. (2019). Lumen apposing metal stents are superior to plastic stents in pancreatic walled-off necrosis: a large international multicenter study. Endosc Int Open.

[81] Zhou X., Lin H., Su X. (2021). Metal versus plastic stents for pancreatic fluid collection drainage: a systematic review and meta-analysis. J Clin Gastroenterol.

[82] Siddiqui A.A., Kowalski T.E., Loren D.E. (2017). Fully covered self-expanding metal stents versus lumen-apposing fully covered self-expanding metal stent versus plastic stents for endoscopic drainage of pancreatic walled-off necrosis: clinical outcomes and success. Gastrointest Endosc.

[83] Chandrasekhara V., Barthet M., Devière J. (2020). Safety and efficacy of lumen-apposing metal stents versus plastic stents to treat walled-off pancreatic necrosis: systematic review and meta-analysis. Endosc Int Open.

[84] Siddiqui A.A., Dewitt J.M., Strongin A. (2013). Outcomes of EUS-guided drainage of debris-containing pancreatic pseudocysts by using combined endoprosthesis and a nasocystic drain. Gastrointest Endosc.

[85] van Santvoort H.C., Besselink M.G., Bakker O.J. (2010). A step-up approach or open necrosectomy for necrotizing pancreatitis. N Engl J Med.

[86] van Brunschot S., Hollemans R.A., Bakker O.J. (2018). Minimally invasive and endoscopic versus open necrosectomy for necrotising pancreatitis: a pooled analysis of individual data for 1980 patients. Gut.

[87] Bakker O.J., van Santvoort H.C., van Brunschot S. (2012). Endoscopic transgastric vs surgical necrosectomy for infected necrotizing pancreatitis: a randomized trial. JAMA.

[88] Bang J.Y., Arnoletti J.P., Holt B.A. (2019). An endoscopic transluminal approach, compared with minimally invasive surgery, reduces complications and costs for patients with necrotizing pancreatitis. Gastroenterology.

[89] Garg P.K., Meena D., Babu D. (2020). Endoscopic versus laparoscopic drainage of pseudocyst and walled-off necrosis following acute pancreatitis: a randomized trial. Surg Endosc.

[90] Varadarajulu S., Bang J.Y., Sutton B.S. (2013). Equal efficacy of endoscopic and surgical cystogastrostomy for pancreatic pseudocyst drainage in a randomized trial. Gastroenterology.

[91] Koop A.H., Palmer W.C., Stancampiano F.F. (2019). Gastric outlet obstruction: a red flag, potentially manageable. Cleve Clin J Med.

[92] Sigmon D.F., Lopez P.P. (2023). StatPearls [Internet].

[93] Ellison E.C., Zollinger R.M. (2016). Zollinger's Atlas of Surgical Operations.

[94] Boghossian M.B., Funari M.P., De Moura D.T.H. (2021). EUS-guided gastroenterostomy versus duodenal stent placement and surgical gastrojejunostomy for the palliation of malignant gastric outlet obstruction: a systematic review and meta-analysis. Langenbecks Arch Surg.

[95] Dawod E., Nieto J.M. (2018). Endoscopic ultrasound guided gastrojejunostomy. Transl Gastroenterol Hepatol.

[96] Krishnamoorthi R., Bomman S., Benias P. (2022). Efficacy and safety of endoscopic duodenal stent versus endoscopic or surgical gastrojejunostomy to treat malignant gastric outlet obstruction: systematic review and meta-analysis. Endosc Int Open.

[97] On W., Huggett M.T., Upasani V. (2022). Endoscopic ultrasound–guided gastrojejunostomy for malignant gastric outlet obstruction in a patient with a previous liver transplant and pancreaticoduodenectomy. ACG Case Rep J.

